# Nuclear respiratory factor 2 induces SIRT3 expression

**DOI:** 10.1111/acel.12360

**Published:** 2015-06-24

**Authors:** F Kyle Satterstrom, William R Swindell, Gaëlle Laurent, Sejal Vyas, Martha L Bulyk, Marcia C Haigis

**Affiliations:** 1 Harvard School of Engineering and Applied SciencesCambridge, MA, 02138, USA; 2 Department of Cell Biology, Harvard Medical SchoolBoston, MA, 02115, USA; 3 Division of Genetics, Department of Medicine, Brigham and Women’s Hospital and Harvard Medical SchoolBoston, MA, 02115, USA; 4 Department of Pathology, Brigham and Women’s Hospital and Harvard Medical SchoolBoston, MA, 02115, USA

**Keywords:** calorie restriction, dietary restriction, microarray analysis, nuclear respiratory factor 2, SIRT3

## Abstract

The mitochondrial deacetylase SIRT3 regulates several important metabolic processes. SIRT3 is transcriptionally upregulated in multiple tissues during nutrient stresses such as dietary restriction and fasting, but the molecular mechanism of this induction is unclear. We conducted a bioinformatic study to identify transcription factor(s) involved in SIRT3 induction. Our analysis identified an enrichment of binding sites for nuclear respiratory factor 2 (NRF-2), a transcription factor known to play a role in the expression of mitochondrial genes, in the DNA sequences of *SIRT3* and genes with closely correlated expression patterns. *In vitro*, knockdown or overexpression of NRF-2 modulated SIRT3 levels, and the NRF-2α subunit directly bound to the *SIRT3* promoter. Our results suggest that NRF-2 is a regulator of SIRT3 expression and may shed light on how SIRT3 is upregulated during nutrient stress.

## Introduction

The NAD^+^-dependent mitochondrial deacetylase sirtuin-3 (SIRT3) is central to the regulation of cellular metabolism, including the adaptation to nutrient stresses such as fasting and dietary restriction (DR) (Lombard *et al*., 2007; Hebert *et al*., 2013). SIRT3 protein levels are upregulated by fasting and DR in liver, where it stimulates fatty acid oxidation (Hirschey *et al*., 2010) and activates key nodes of ketone body production (Shimazu *et al*., 2010) and the urea cycle (Hallows *et al*., 2011). SIRT3 mRNA levels are also upregulated by DR in brown adipose tissue, where SIRT3 activates mitochondrial thermogenesis (Shi *et al*., 2005). In addition, SIRT3 mediates some of the beneficial effects of DR, such as the activation of mitochondrial superoxide dismutase to reduce oxidative stress (Qiu *et al*., 2010; Tao *et al*., 2010) and the prevention of age-related hearing loss in mice (Someya *et al*., 2010). Conversely, livers of mice fed a chronic high-fat diet exhibit reduced SIRT3 mRNA and protein levels (Hirschey *et al*., 2011), indicating that SIRT3 expression is dynamically regulated by nutrient intake.

Surprisingly, little is known about the molecular control of SIRT3 expression. In murine adipocytes and hepatocytes, the transcription factor estrogen-related receptor α (ERRα) has been shown to induce SIRT3 expression in conjunction with peroxisome proliferator-activated receptor γ coactivator 1-α (PGC-1α) (Kong *et al*., 2010; Giralt *et al*., 2011). In this study, we employed a bioinformatic approach to identify additional transcription factors which regulate SIRT3 expression. We searched publicly available microarray data to identify datasets with an induction of SIRT3 by either DR or fasting and then computationally identified transcription factor binding motifs enriched in the regulatory regions of *SIRT3* and co-induced genes. Our bioinformatic analysis and experimental validation in cell culture identified nuclear respiratory factor 2 (NRF-2) as a novel transcriptional regulator of SIRT3 expression.

## Results

### Bioinformatic analysis

**Fig 1:**
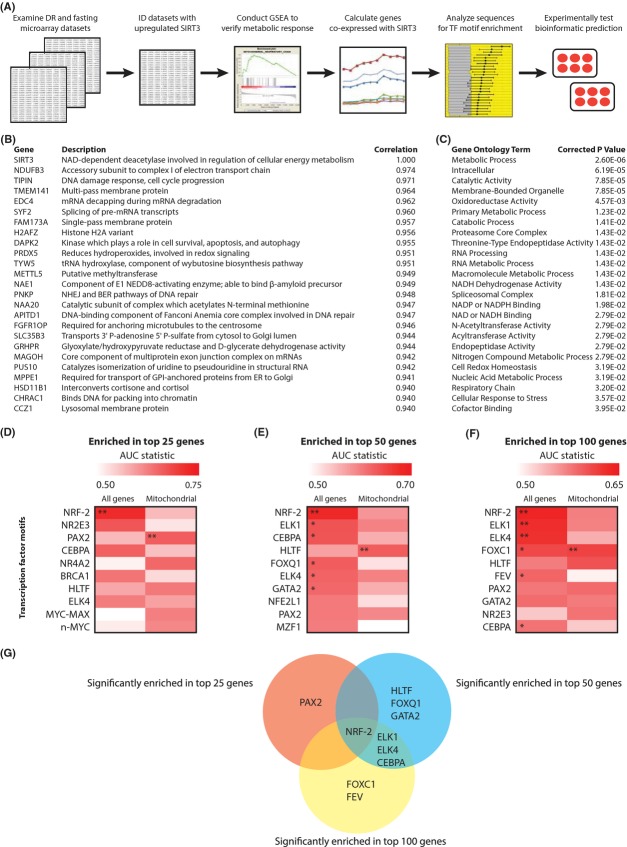
Bioinformatic identification of NRF-2 binding site enrichment in DNA sequences of *SIRT3* and co-expressed genes. (A) Overview of bioinformatic steps analyzing transcription factor binding motif enrichment in the DNA sequences of *SIRT3* and co-expressed genes. (B) Top 25 most SIRT3-correlated genes (by Pearson’s *r*) in the mouse neocortex dataset. (C) Enrichment of selected gene ontology terms in top 50 most SIRT3-correlated genes in neocortex (full results in Table S2, Supporting information). (D–F) Heat maps of transcription factor motif enrichment in the (D) 25 most SIRT3-correlated genes, (E) 50 most SIRT3-correlated genes, and (F) 100 most SIRT3-correlated genes (by expression levels across samples) for the neocortex dataset. All genes = most SIRT3-correlated genes analyzed from all genes in dataset. Mitochondrial = most SIRT3-correlated mitochondrial genes analyzed in the dataset. Top ten motifs are shown, ordered by motif’s maximum AUC score, a measure of enrichment. Red = greater enrichment; white = less enrichment. * indicates *q* < 0.05; ** indicates *q* < 0.01. (G) Overlap of significantly enriched transcription factor motifs identified in (D–F), showing results from analyzing the 25 (red circle), 50 (blue circle), and 100 (yellow circle) most SIRT3-correlated genes for the neocortex dataset.

To identify transcription factors involved in SIRT3 induction, we undertook a systematic bioinformatic approach (Fig.1A). We first identified datasets in which SIRT3 mRNA expression was increased with DR in neocortex (GSE11291; Barger *et al*., 2008), cochlea (GSE4786, Someya *et al*., 2007), and liver (GSE26267, Streeper *et al*., 2012), and with fasting in kidney (GSE24504, Hakvoort *et al*., 2011). We conducted gene set enrichment analysis (Table S1, Supporting information) to verify that this induction was part of a larger metabolic adaptation. Next, based on the rationale that co-expressed genes may share common transcriptional regulators, we identified the genes most closely co-induced with SIRT3 in each dataset (Fig.1B, File S1, Supporting information). Groups of 25, 50, and 100 genes were analyzed, allowing for greater statistical power than an analysis of SIRT3 alone. Gene ontology analysis showed that the genes most closely co-induced with SIRT3 were enriched in metabolism-related annotations, as well as processes such as the cellular response to stress (Fig.1C, Table S2, Supporting information), suggesting that many co-regulated genes are functionally related to SIRT3. For each dataset, a DNA sequence analysis algorithm (Warner *et al*., 2008) was then used to calculate the enrichment of a set of transcription factor motifs in 20 kbp of sequence surrounding (i) the most SIRT3-correlated genes overall or (ii) the most SIRT3-correlated mitochondrial genes (as determined by inclusion in the MitoCarta, Pagliarini *et al*., 2008; File S2, Supporting information). The mitochondrial group was included because SIRT3 is a mitochondrial-localized protein, and factors which regulate its expression may act specifically on nuclear-encoded mitochondrial genes during processes induced by nutrient stress such as mitochondrial biogenesis (e.g., Scarpulla, 2002).

The highest-scoring transcription factor motif identified by this study was nuclear respiratory factor 2 (NRF-2, also known as GABP) (Rosmarin *et al*., 2004) from the neocortex dataset, regardless of number of genes analyzed (Fig.1D–F; for the other datasets, Fig. S1 (Supporting information); full results for all datasets are in File S3, Supporting information). NRF-2 was also the only motif significantly enriched across the analyses of 25, 50, and 100 genes (Fig.1G). NRF-2 is an E26 transformation-specific (ETS) family transcription factor that is important for the expression of many mitochondrial genes (Scarpulla, 2002). NRF-2 is bound and co-activated by PGC-1α, and it is central to mitochondrial biogenesis and metabolism (Mootha *et al*., 2004; Baldelli *et al*., 2013). All ten nuclear-encoded cytochrome *c* oxidase subunits have functional NRF-2 binding sites (Ongwijitwat & Wong-Riley, 2005), and recognition sites for NRF-2 are also present in the promoters of ATP synthase subunit-β and succinate dehydrogenase subunits B, C, and D (Scarpulla, 2002). The effects of DR/fasting on NRF-2 and its targets in the datasets studied are included as Fig. S2 (Supporting information). SIRT3 directly interacts with several components of the electron transport chain, including ATP synthase subunit β and succinate dehydrogenase subunit A (Finley *et al*., 2011; Vassilopoulos *et al*., 2014), and one of its few known regulators is PGC-1α. This functional overlap supports our bioinformatic finding of NRF-2 as a candidate regulator of *SIRT3*.

### Analysis of SIRT3 promoter

**Fig 2:**
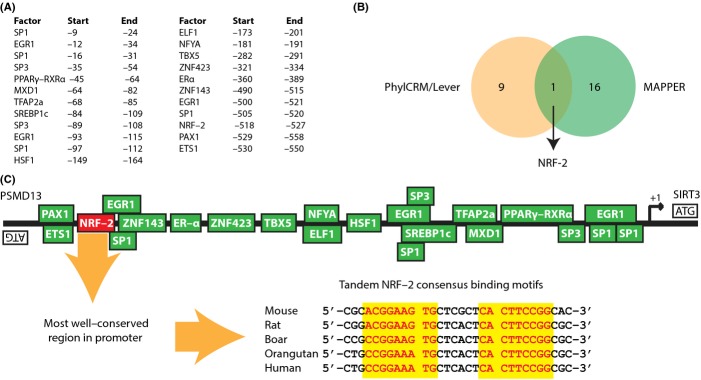
Analysis of *SIRT3* promoter. (A) List of transcription factor binding sites identified by MAPPER in the mouse *SIRT3* promoter. (B) Overlap of enriched transcription factor motifs identified by analysis of SIRT3 correlated genes in the neocortex dataset (pink circle) with transcription factors identified in the *SIRT3* promoter (green circle). (C) Schematic of *PSMD13*-*SIRT3* promoter, highlighting tandem NRF-2 binding sites with red text/yellow background and showing sequence across multiple species.

To investigate whether NRF-2 regulates SIRT3 expression, we probed for NRF-2 binding sites in the *SIRT3* promoter. *SIRT3* shares a short bidirectional promoter with the 26S proteasome non-ATPase regulatory subunit 13 (PSMD13) (Bellizzi *et al*., 2007). The two genes are coded on opposite strands, with their 5′ ends toward each other and < 1 kbp apart. Because of the bidirectional promoter, any binding sites in the *SIRT3* promoter are also in the *PSMD13* promoter. Dissection of the promoter using a separate sequence analysis tool (MAPPER, http://genome.ufl.edu/mapper/; Marinescu *et al*., 2005) identified several transcription factor binding motifs (Fig.2A). Notably, NRF-2 was the only overlap between our significant DNA sequence analysis results for the neocortex dataset and the MAPPER results (Fig.2B). Moreover, NRF-2 sites often occur in tandem (Virbasius & Scarpulla, 1991), and the best-conserved region of the entire promoter is a pair of NRF-2 consensus sequences (Fig.2C). NRF-2 is also known to direct transcription from many bidirectional promoters (Collins *et al*., 2007). Taken together with the motif enrichment results above, our findings strongly suggested that NRF-2 may play a role in regulating SIRT3 expression, and perhaps PSMD13 expression as well, via the *PSMD13*-*SIRT3* promoter.

### Experimental investigation of NRF-2 and SIRT3

**Fig 3:**
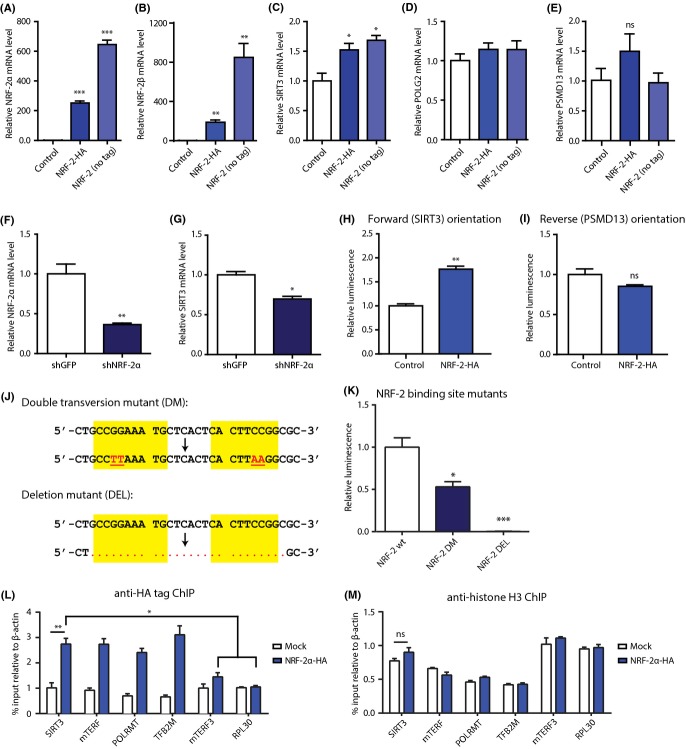
Experimental investigation of NRF-2 control of the *SIRT3* promoter. (A) Validation of NRF-2α overexpression and B) NRF-2β1 overexpression in 293T cells by quantitative PCR. The two subunits together compose NRF-2. (C) Effect of NRF-2 overexpression on mRNA levels of SIRT3, (D) mitochondrial DNA polymerase subunit γ-2 (POLG2), and (E) PSMD13. (F) Validation of NRF-2α knockdown in 293T cells and (G) effect on SIRT3 mRNA levels. (H) Relative luminescence from forward (SIRT3) and (I) reversed (PSMD13) bidirectional promoter luciferase reporter following the overexpression of vector control or NRF-2-HA in 293T cells. (J) Diagrams of changes introduced in two reporter constructs with mutated NRF-2 binding sites: a double transversion mutation (DM) and a 26-base pair deletion mutation (DEL). Mutations introduced are shown in red. NRF-2 binding sites are highlighted with a yellow background. (K) Relative luminescence from SIRT3 promoter reporter with wild-type sequence (NRF-2 wt), mutated NRF-2 binding sites (NRF-2 DM), or deleted NRF-2 binding sites (NRF-2 DEL). (L) Chromatin immunoprecipitation of HA tag in 293T cells following the overexpression of NRF-2α-HA, showing percent input (relative to β-actin) of SIRT3 and positive (mTERF, POLRMT, TFB2M) and negative (mTERF3, RPL30) NRF-2 controls. (M) Chromatin immunoprecipitation of histone H3 in 293T cells following the overexpression of NRF-2α-HA, showing percent input (relative to β-actin) of SIRT3 and positive and negative NRF-2 controls. For A–G, *n* = 3 samples per condition, and B2M was used as the reference gene; for H–K, *n* = 4 samples per condition; for L and M, *n* = 3 separate immunoprecipitations; and for all, bars are standard error. * indicates *P* < 0.05; ** indicates *P* < 0.01; and *** indicates *P* < 0.001. Two-tailed Student’s *t*-test was used for *P* values.

We tested experimentally whether NRF-2 regulates SIRT3 and PSMD13 gene expression using human 293T cells. NRF-2 functions as a heterodimer, with the α subunit binding DNA and the β subunit facilitating binding between heterodimers (Batchelor *et al*., 1998). When NRF-2α and NRF-2β1 were transiently overexpressed together (Fig.3A,B), SIRT3 mRNA levels were significantly induced (*P* = 0.046 for HA-tagged NRF-2, *P* = 0.003 for untagged NRF-2, Fig.3C) to a greater degree than known NRF-2 target DNA polymerase subunit γ-2 (POLG2) (*P* = 0.26 for HA-tagged NRF-2, *P* = 0.44 for untagged NRF-2, Fig.3D). PSMD13 levels were not significantly affected (*P* = 0.21 for HA-tagged NRF-2, *P* = 0.77 for untagged NRF-2, Fig.3E) and were likewise only weakly correlated with SIRT3 expression in the four datasets examined (File S1, Supporting information). Conversely, when the DNA-binding NRF-2α subunit was knocked down (Fig.3F), SIRT3 mRNA levels significantly dropped (*P* = 0.005, Fig.3G). These data demonstrate that SIRT3 expression responded dynamically to NRF-2 levels.

Having identified the presence of canonical NRF-2 binding sites in the *SIRT3* promoter and characterized the response of SIRT3 expression to NRF-2 overexpression, we next tested whether the response to NRF-2 occurs via the *SIRT3* promoter. A luciferase reporter plasmid driven by the shared promoter in either the SIRT3 or PSMD13 direction (Satterstrom & Haigis, 2014) was transfected into 293T cells. NRF-2 was then overexpressed and luminescence measured. Overexpression of NRF-2 increased the activation of the reporter when driven by the *SIRT3* promoter (*P* < 0.01, Fig.3H) but did not have a significant effect when the promoter was inserted in the PSMD13 direction (activity decreased, *P* = 0.09, Fig.3I). Further, point mutation or deletion of the NRF-2 binding site greatly reduced the activity of the reporter in the SIRT3 direction (Fig.3J–K). Together with the quantitative PCR data, these data suggest that NRF-2 may control SIRT3 expression by direct interaction with the *SIRT3* promoter.

To test physical binding of the *SIRT3* promoter by NRF-2, a chromatin immunoprecipitation was performed in 293T cells transiently overexpressing HA-tagged NRF-2α, the DNA-binding subunit of the NRF-2 heterodimer. Following chromatin isolation, HA tag was immunoprecipitated and qPCR was used to quantify the levels of target DNA. Immunoprecipitation and quantification were carried out three times. Using β-actin as a background normalization factor across experiments, *SIRT3* was significantly enriched in the NRF-2α-treated condition compared to the untreated condition (*P* = 0.005). This enrichment was to approximately the same degree as genes known to be transcriptionally regulated by NRF-2 (Bruni *et al*., 2010), such as mitochondrial transcription termination factor (*mTERF*) (*P* = 0.99), mitochondrial RNA polymerase (POLRMT) (*P* = 0.30), and mitochondrial transcription factor B2 (TFB2M) (*P* = 0.43), and to a significantly greater degree than non-NRF-2-regulated genes such as *mTERF3* (*P* = 0.01) and ribosomal protein L30 (*RPL30*) (*P* = 0.002) (Fig.3L). This enhancement was not seen with control anti-histone H3 immunoprecipitations (Fig.3M). These results suggest that NRF-2α physically binds the *SIRT3* promoter to affect SIRT3 gene expression.

## Discussion

In this study, we have discovered that nuclear respiratory factor 2 (NRF-2) is a novel regulator of SIRT3 expression. We used a bioinformatic analysis to show that NRF-2 binding sites are highly enriched in the regulatory regions of SIRT3 and genes that are similarly induced by DR. We have also demonstrated that SIRT3 mRNA levels respond to overexpression or knockdown of NRF-2 in 293T cells and that the same effect occurs when using a luciferase reporter with the *SIRT3* promoter. Finally, we have shown by chromatin immunoprecipitation that the α subunit of NRF-2 binds the *SIRT3* promoter directly, suggesting a model wherein NRF-2 binds the *SIRT3* promoter, leading to the expression of SIRT3 mRNA. Our data also suggest that *SIRT3* and *PSMD13* are regulated independently, as NRF-2 induces the expression of SIRT3 but not PSMD13 under the conditions studied.

NRF-2 binds and is co-activated by PGC-1α, leading to an increase in its induction of target genes (Mootha *et al*., 2004; Baldelli *et al*., 2013). Notably, ERRα, which is already known to play a role in activating SIRT3 transcription, is also co-activated by PGC-1α (Schreiber *et al*., 2003). Both NRF-2 and ERRα drive the expression of oxidative genes as well as each other (Mootha *et al*., 2004), but they may be active at different times or in different tissues; in support of this idea, NRF-2 was a significant result for our analysis of the neocortex dataset, while one significant result for our analysis of the liver dataset was ERRβ (which has a nearly identical binding motif to ERRα; ERRα was not in the set of JASPAR motifs). Although further study is needed to determine the relative importance of these transcription factors for the induction of SIRT3 in different physiological contexts, our data suggest that NRF-2 plays an important role.

Because PGC-1α is induced in certain tissues by fasting or DR (Lehman *et al*., 2000), the PGC-1α/NRF-2 pathway may underlie the upregulation of SIRT3 and other mitochondrial genes in DR. The overlap in pathways affected by NRF-2 and SIRT3 supports this idea. Even under basal conditions, the deletion of NRF-2 in mouse embryonic fibroblasts reduces many markers of mitochondrial biogenesis, including oxygen consumption and ATP production (Yang *et al*., 2014). SIRT3, meanwhile, is known to increase oxygen consumption (Shi *et al*., 2005), to be important for ATP production (Ahn *et al*., 2008), and to be important for mitochondrial biogenesis (Kong *et al*., 2010). These functions are enhanced during stress (e.g., Vassilopoulos *et al*., 2014). Thus, not only do NRF-2 and SIRT3 function in similar stress-induced pathways, but SIRT3 carries out functions which are abrogated when NRF-2 is absent. Although whole-body knockout of NRF-2 leads to embryonic lethality (Ristevski *et al*., 2004), necessitating the use of tissue-specific deletion or other more nuanced methods, further study will determine whether NRF-2 is required for the action of SIRT3 under basal and stressed conditions.

Our analysis additionally identified multiple transcription factors of interest which may be involved in the regulation of SIRT3 expression. CCAAT/enhancer-binding protein α (CEBPα), whose motif was enriched in the neocortex dataset, and c-MYC, whose motif was enriched in the kidney dataset, regulate metabolic processes and would be reasonable candidate regulators of SIRT3. CEBPα regulates transcription of the human fat mass and obesity-associated gene (FTO) (Ren *et al*., 2014), and c-MYC is well known for its role in cancer metabolism (reviewed in Miller *et al*., 2012). Additionally, our inspection of the *SIRT3* promoter identified binding sites for transcription factors known to interact with NRF-2, including Sp1 and Sp3 (Galvagni *et al*., 2001), or share common targets with it, including ZNF143 (Gérard *et al*., 2007) and EGR1 (Fromm & Rhode, 2004). Finally, ETS-1, ELF-1, ELK1, and ELK4 are all members of the same family of transcription factors as NRF-2. It is possible that one or more was identified because of its similar binding motif without actually playing a role in SIRT3 expression; it is also possible that they are important in different contexts, or, when they are co-expressed, multiple factors may bind the same promoter element to activate gene expression with different strengths (Takahashi *et al*., 2008).

Our findings are an important step toward elucidating the molecular regulation upstream of SIRT3 expression. SIRT3 levels are increased in multiple tissues during nutrient stresses such as DR. DR is associated with increased lifespan (Anderson & Weindruch, 2010), as is a *SIRT3* allele with increased activity (Bellizzi *et al*., 2005). We have shown that NRF-2 plays a role in the induction of SIRT3, and this may help to uncover the molecular pathways activated by DR and to inform therapies that delay the onset of age-related disease.

## Experimental procedures

### Analysis of SIRT3 levels in microarray datasets

Microarray series data and corresponding platform annotations from DR/fasting experiments were downloaded from the Gene Expression Omnibus at http://www.ncbi.nlm.nih.gov/geo/. Significance of the effect on SIRT3 was determined using a two-tailed Student’s *t*-test with a significance threshold of *P* < 0.05. In cases where the platform had more than one probe for SIRT3, each probe was examined individually.

### Gene set enrichment analysis

Gene set enrichment analysis of each dataset showing a significant induction of SIRT3 by DR/fasting was performed using the GSEA version 2.0.10 java program from http://www.broadinstitute.org/gsea/downloads.jsp. Data files were prepared with the Broad GEOImporter preprocess utility and were analyzed for the enrichment of gene ontology-related gene sets as contained in the c5.all.v3.1.symbols.gmt gene sets database. Datasets were collapsed from probes to gene symbols using default settings, and 1000 permutations were conducted. Gene set permutations were used because none of the datasets had a sufficient number of samples per condition to allow the use of phenotype permutations. Datasets which showed no significant induction of any gene sets were not included in subsequent analysis.

### Calculation of co-regulated gene sets

The robust multiarray average (RMA) algorithm as implemented in the Bioconductor package ‘affy’ (Gautier *et al*., 2004) for R was used to background adjust and normalize the raw data files for each dataset that showed upregulation of Sirt3 and gene ontology-related gene sets upon DR (except for GSE24504, for which the GEO series matrix was used). Correlations (Pearson’s *r*) were then computed between the SIRT3 probe and each probe in the array. To control for probe specificity, probes whose label contained an _s_ or _x_ were removed from datasets generated from the Affymetrix Mouse Genome 430 2.0 Array unless doing so would leave the gene without any valid probes. For genes with multiple probes, correlations were averaged to compute a single correlation value for the gene.

### Gene ontology analysis

Analysis of overrepresented gene ontology terms was carried out within the Cytoscape software program using the BiNGO plugin (Maere *et al*., 2005), with the whole *mus musculus* annotation as the reference set. The hypergeometric statistical test and Benjamini & Hochberg FDR correction options were used.

### DNA sequence analysis

Analysis was conducted using the PhylCRM-Lever algorithm (Warner *et al*., 2008). The sequence analysis looked at 20 kbp for each gene, from −10 kbp to +10 kbp surrounding the transcription start site. Motif enrichment was calculated for all 130 mammalian motifs hosted by version 4 of the JASPAR database (Portales-Casamar *et al*., 2010; http://jaspar.genereg.net/). The analysis included weighting based on the conservation of transcription factor motifs across genomes of multiple species: mouse (mm9), rat (rn4), human (hg18), chimpanzee (panTro2), rhesus macaque (rheMac2), cow (bosTau3), dog (canFam2), and chicken (galGal3). SIRT3 itself was included in the lists of SIRT3-correlated genes.

Additional DNA sequence analysis was performed using MAPPER (Marinescu *et al*., 2005). MAPPER database runs with default filtering options examined 2 kbp of *mus musculus* DNA sequence upstream of the transcription start, using TRANSFAC, MAPPER, and JASPAR models.

### Cell culture

Human embryonic kidney 293T cells were grown in Dulbecco’s modified Eagle’s minimal essential medium (Life Technologies, cat. # 11995) with 10% fetal bovine serum (HyClone GE Healthcare, Little Chalfont, Buckinghamshire, United Kingdom) and 1% penicillin–streptomycin supplement (Life Technologies, Carlsbad, CA, USA) and maintained in an incubator at standard tissue culture conditions (37 °C, 5% CO_2_). A control knockdown line was created using a GFP shRNA construct, and a 293T NRF-2α knockdown cell line was created using shRNA construct TRCN0000235698 from the RNAi consortium (both via the Dana-Farber/Harvard Cancer Center RNAi Core facility).

### Expression and reporter plasmids

HA-tagged overexpression plasmids for NRF-2α and NRF-2β1 were generated using Gateway cloning techniques, starting from the HsCD00080063 and HsCD00370955 entry clones, respectively, from the PlasmID database of the Dana-Farber/Harvard Cancer Center DNA Resource Core. Untagged overexpression plasmids were generated via Gateway cloning techniques from HsCD00296808 and HsCD00338810. The reversible SIRT3-PSMD13 promoter reporter construct was cloned as described (Satterstrom & Haigis, 2014). Plasmid DNA was transfected into 293T cells using FuGene6 (Roche, Basel, Switzerland) according to the manufacturer’s instructions, and cells were allowed to grow for 48 h prior to analysis.

### SIRT3 promoter reporter mutagenesis

SIRT3 promoter reporter mutants were cloned with altered NRF-2 binding sites (a change of two bases in each of the two binding sites) or with deleted NRF-2 binding sites (both binding sites and the intervening sequence removed, a total of 26 bases). A QuikChange II mutagenesis kit (Agilent Technologies, Santa Clara, CA) was used to produce the mutant with altered binding sites, in which both NRF-2 binding sites have a mutation of CCGGAA to CCTTAA (Virbasius *et al*., 1993). To produce the mutant with both binding sites deleted, a Q5 Site-Directed Mutagenesis Kit (New England Biolabs, Ipswich, MA, USA) was used with an annealing temperature of 72 °C. Primers used are given in Table S3 (Supporting information). For the altered binding site mutant, the pair labeled DM1 was used prior to the pair labeled DM2.

### Luciferase

Cells were grown in an opaque 96-well plate. Following co-transfection of the SIRT3 reporter plasmid and the pRL renilla control vector (Promega, Madison, WI, USA), the Dual-Luciferase Reporter Assay System (Promega) was used according to the manufacturer’s instructions. Sample luminescence was assayed with a Cary Varian Eclipse fluorescence spectrophotometer.

### Chromatin immunoprecipitation

Chromatin immunoprecipitation (ChIP) was performed with the SimpleChIP Enzymatic Chromatin IP Kit (Cell Signaling, Danvers, MA, USA) with antibodies against HA tag (Cell Signaling), histone H3 (Cell Signaling), or normal rabbit IgG (Cell Signaling). Relative quantities of precipitated DNA fragments were obtained using quantitative PCR. Three separate immunoprecipitations were performed. To control for variation in percent input across precipitations, the percent input of each target gene was divided by the percent input of a random background gene, β-actin. These normalized values were then averaged across precipitations and analyzed for significance.

### Quantitative PCR

For overexpression experiments in cells, RNA was extracted using RNEasy Mini Kits (Qiagen, Hilden, Germany) and cDNA was synthesized using iScript cDNA Synthesis Kits (Bio-Rad, Hercules, CA, USA). For both overexpression experiments and ChIP analysis, quantitative PCR was performed with 2× PerfeCTa SYBR Green FastMix (Quanta BioSciences, Gaithersburg, MD, USA). Control primers for ChIP analysis were from Bruni *et al*. (2010); other primer sequences are given in Table S3 (Supporting information) (except RPL30; Cell Signaling). For non-ChIP analysis, β2-microglobulin (B2M) was used as a reference gene.

## Funding

F. K. S. was supported by NIH Training Grant No. T32 DK007260. M. L. B. was supported in part by NIH Grant No. R01 DK088718. M. C. H. was supported by an American Cancer Society Research Scholar Award and the Glenn Foundation for Medical Research.

## Conflict of interest

None declared.

## Supporting Information

**Fig. S1** Heat maps of transcription factor motif enrichment in DNA sequences of *SIRT3* and co-expressed genes for cochlea (A–C), liver (D–F), and kidney (G–I) datasets based on sequence analysis of the top 25, 50, and 100 SIRT3-correlated genes in each dataset.

**Fig. S2** Effect of DR or fasting on NRF-2 and its target genes, including ERRα, in the datasets used in this study (neocortex, GSE11291; cochlea, GSE4786; liver, GSE26267, kidney, GSE24504) as well as a mouse dataset previously generated by our laboratory (GSE34773, skeletal muscle).

**Table S1** Gene set enrichment analysis summary for datasets with significant induction of SIRT3 in mouse tissue by dietary restriction or fasting. **Table S2** Overrepresented gene ontology terms in the top 50 SIRT3-correlated genes in each of the four datasets analyzed. **Table S3** Sequences of primers used in this study.

**File S1** Complete correlations with SIRT3 expression for each gene in each of four datasets analyzed.

**File S2** All input gene groups used in PhylCRM-Lever DNA sequence analysis algorithm.

**File S3** Complete PhylCRM-Lever results for each analyzed dataset.
