# Supplementary figures and images for: Nuclear respiratory factor 2 induces SIRT3 expression
Source: Aging Cell. 2015 Jun 24;14(5):818–25. doi: 10.1111/acel.12360 (PMC4568969; doi:10.1111/acel.12360)

Figure S2

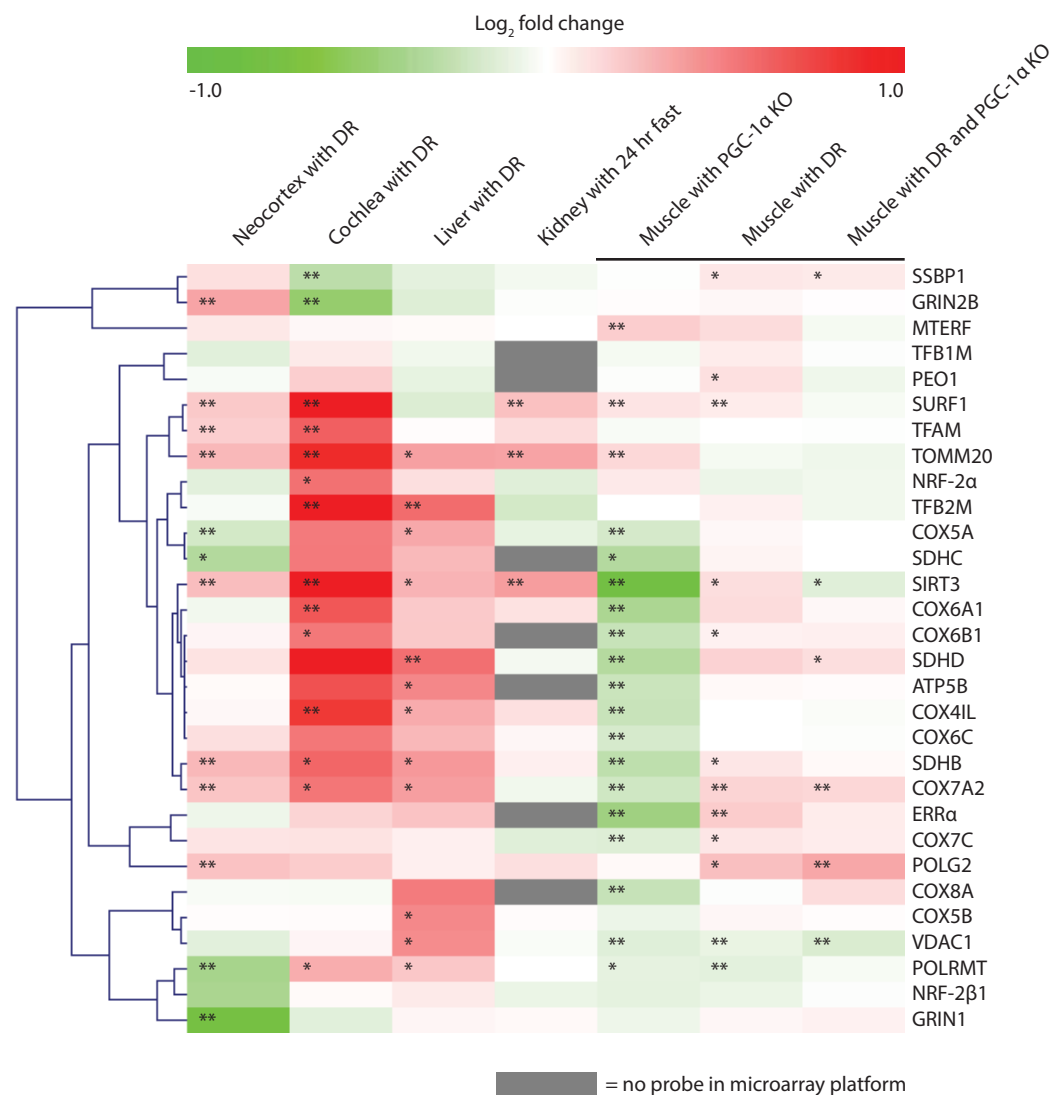

Supplement: Supplementary file 2 [file acel0014-0818-sd2.pdf]
