# Supplementary material for: Nuclear respiratory factor 2 induces SIRT3 expression
Source: Aging Cell. 2015 Jun 24;14(5):818–25. doi: 10.1111/acel.12360 (PMC4568969; doi:10.1111/acel.12360)
Supplement: Supplementary file 3 [file acel0014-0818-sd3.docx]

**Table S1.** Gene set enrichment analysis summary for datasets with significant induction of SIRT3 in mouse tissue by dietary restriction or fasting. The FDR rankings for the kidney dataset are an average across four time points.

| **Dataset** | **# gene sets with FDR < 0.05 and nominal pval < 0.01** | **Top ten enriched gene sets with CR (by FDR)** |
| --- | --- | --- |
| Neocortex | 9 | Replication Fork  Structural Constituent of Ribosome  Ribonuclease Activity  Neuropeptide Hormone Activity  Organellar Ribosome  Mitochondrial Respiratory Chain  Proton Transporting Two Sector ATPase Complex  Endoribonuclease Activity  Cellular Respiration  Endonuclease Activity |
| Cochlea | 72 | Ligase Activity  Protein Folding  Microbody Part  mRNA Binding  GTPase Activity  Peroxisomal Part  Structural Constituent of Ribosome  Phosphatase Inhibitor Activity  Organellar Ribosome  Mitochondrial Ribosome |
| Liver | 21 | Mitochondrial Membrane Part  Mitochondrial Membrane  Organelle Inner Membrane  Mitochondrial Inner Membrane  Mitochondrial Envelope  Mitochondrial Part  Mitochondrion  Structural Constituent of Ribosome  Cellular Respiration  Envelope |
| Kidney | 12 hrs: 10  24 hrs: 9  48 hrs: 7  72 hrs: 3 | Fatty Acid Metabolic Process  Fatty Acid Oxidation  Monocarboxylic Acid Metabolic Process  Cellular Lipid Catabolic Process  Solute Sodium Symporter Activity  Peroxisomal Part  Lipid Catabolic Process  Microbody Part  Microbody  Peroxisome |

**Table S2.** Overrepresented gene ontology terms in the top 50 SIRT3-correlated genes in each of the four datasets analyzed. Corr P value = corrected P value. # in set refers to number of the 50 that had the annotation; # overall refers to the number of genes with that annotation overall. For the neocortex, cochlea, liver, and kidney datasets, annotations were retrieved for 50, 50, 48, and 50 genes, respectively, with the reference set containing 30,010, 30,009, 30,011, and 30,009 genes. Results with only one gene in the test set are not shown. Analysis carried out by BinGO plugin in Cytoscape.

| **Dataset** | **GO Term** | **GO ID** | **P value** | **Corr P value** | **# in set** | **# overall** |
| --- | --- | --- | --- | --- | --- | --- |
| **Neocortex** | metabolic process | 8152 | 4.38E-09 | 2.60E-06 | 30 | 6470 |
|  | intracellular part | 44424 | 1.62E-07 | 4.82E-05 | 34 | 9546 |
|  | intracellular | 5622 | 3.12E-07 | 6.19E-05 | 34 | 9782 |
|  | cellular process | 9987 | 5.58E-07 | 7.85E-05 | 32 | 8926 |
|  | catalytic activity | 3824 | 7.36E-07 | 7.85E-05 | 23 | 4858 |
|  | intracellular membrane-bounded organelle | 43231 | 9.03E-07 | 7.85E-05 | 28 | 7109 |
|  | membrane-bounded organelle | 43227 | 9.37E-07 | 7.85E-05 | 28 | 7121 |
|  | cellular metabolic process | 44237 | 1.06E-06 | 7.85E-05 | 24 | 5371 |
|  | cell part | 44464 | 2.38E-06 | 1.42E-04 | 40 | 14254 |
|  | cell | 5623 | 2.39E-06 | 1.42E-04 | 40 | 14255 |
|  | intracellular organelle | 43229 | 3.62E-06 | 1.92E-04 | 29 | 8080 |
|  | organelle | 43226 | 3.87E-06 | 1.92E-04 | 29 | 8105 |
|  | cytoplasm | 5737 | 5.22E-06 | 2.39E-04 | 26 | 6765 |
|  | nucleus | 5634 | 1.83E-05 | 7.79E-04 | 19 | 4148 |
|  | oxidation reduction | 55114 | 8.75E-05 | 3.47E-03 | 7 | 640 |
|  | oxidoreductase activity | 16491 | 1.23E-04 | 4.57E-03 | 7 | 676 |
|  | primary metabolic process | 44238 | 3.53E-04 | 1.23E-02 | 20 | 5587 |
|  | catabolic process | 9056 | 4.28E-04 | 1.41E-02 | 7 | 830 |
|  | proteasome core complex | 5839 | 4.57E-04 | 1.43E-02 | 2 | 19 |
|  | macromolecule metabolic process | 43170 | 5.01E-04 | 1.43E-02 | 17 | 4401 |
|  | RNA metabolic process | 16070 | 5.39E-04 | 1.43E-02 | 6 | 616 |
|  | threonine-type endopeptidase activity | 4298 | 5.60E-04 | 1.43E-02 | 2 | 21 |
|  | threonine-type peptidase activity | 70003 | 5.60E-04 | 1.43E-02 | 2 | 21 |
|  | RNA processing | 6396 | 5.94E-04 | 1.43E-02 | 5 | 410 |
|  | cellular catabolic process | 44248 | 6.32E-04 | 1.43E-02 | 6 | 635 |
|  | NADH dehydrogenase (ubiquinone) activity | 8137 | 6.73E-04 | 1.43E-02 | 2 | 23 |
|  | NADH dehydrogenase (quinone) activity | 50136 | 6.73E-04 | 1.43E-02 | 2 | 23 |
|  | NADH dehydrogenase activity | 3954 | 6.73E-04 | 1.43E-02 | 2 | 23 |
|  | oxidoreductase activity, acting on NADH or NADPH, quinone or similar compound as acceptor | 16655 | 8.62E-04 | 1.76E-02 | 2 | 26 |
|  | mRNA processing | 6397 | 8.88E-04 | 1.76E-02 | 4 | 257 |
|  | spliceosomal complex | 5681 | 9.42E-04 | 1.81E-02 | 3 | 115 |
|  | NADP or NADPH binding | 50661 | 1.07E-03 | 1.98E-02 | 2 | 29 |
|  | cellular macromolecule metabolic process | 44260 | 1.27E-03 | 1.98E-02 | 15 | 3902 |
|  | mRNA metabolic process | 16071 | 1.48E-03 | 1.98E-02 | 4 | 295 |
|  | endopeptidase activity | 4175 | 2.66E-03 | 2.79E-02 | 4 | 347 |
|  | coenzyme binding | 50662 | 2.69E-03 | 2.79E-02 | 3 | 166 |
|  | cellular nitrogen compound metabolic process | 34641 | 2.74E-03 | 2.79E-02 | 12 | 2946 |
|  | N-acetyltransferase activity | 8080 | 2.80E-03 | 2.79E-02 | 2 | 47 |
|  | NAD or NADH binding | 51287 | 2.92E-03 | 2.79E-02 | 2 | 48 |
|  | oxidoreductase activity, acting on NADH or NADPH | 16651 | 2.92E-03 | 2.79E-02 | 2 | 48 |
|  | binding | 5488 | 3.27E-03 | 2.79E-02 | 27 | 10293 |
|  | acyltransferase activity | 8415 | 3.33E-03 | 2.79E-02 | 3 | 179 |
|  | intracellular organelle part | 44446 | 3.42E-03 | 2.79E-02 | 11 | 2626 |
|  | transferase activity, transferring acyl groups other than amino-acyl groups | 16747 | 3.43E-03 | 2.79E-02 | 3 | 181 |
|  | nitrogen compound metabolic process | 6807 | 3.47E-03 | 2.79E-02 | 12 | 3031 |
|  | transferase activity, transferring acyl groups | 16746 | 3.76E-03 | 2.99E-02 | 3 | 187 |
|  | nuclear part | 44428 | 3.84E-03 | 3.00E-02 | 6 | 907 |
|  | cell redox homeostasis | 45454 | 4.24E-03 | 3.19E-02 | 2 | 58 |
|  | organelle part | 44422 | 4.26E-03 | 3.19E-02 | 11 | 2702 |
|  | RNA splicing | 8380 | 4.29E-03 | 3.19E-02 | 3 | 196 |
|  | nucleic acid metabolic process | 90304 | 4.37E-03 | 3.19E-02 | 10 | 2318 |
|  | proteasome complex | 502 | 4.38E-03 | 3.19E-02 | 2 | 59 |
|  | nucleobase, nucleoside, nucleotide and nucleic acid metabolic process | 6139 | 4.45E-03 | 3.19E-02 | 11 | 2717 |
|  | acetyltransferase activity | 16407 | 4.53E-03 | 3.19E-02 | 2 | 60 |
|  | N-acyltransferase activity | 16410 | 4.83E-03 | 3.19E-02 | 2 | 62 |
|  | cellular macromolecule catabolic process | 44265 | 5.06E-03 | 3.20E-02 | 3 | 208 |
|  | respiratory chain | 70469 | 5.13E-03 | 3.20E-02 | 2 | 64 |
|  | transferase activity | 16740 | 5.17E-03 | 3.20E-02 | 8 | 1628 |
|  | ribonucleoprotein complex | 30529 | 5.79E-03 | 3.55E-02 | 4 | 432 |
|  | cellular response to stress | 33554 | 5.88E-03 | 3.57E-02 | 4 | 434 |
|  | cofactor binding | 48037 | 6.84E-03 | 3.95E-02 | 3 | 232 |
| **Dataset** | **GO Term** | **GO ID** | **P value** | **Corr P value** | **# in set** | **# overall** |
| **Cochlea** | cytoplasm | 5737 | 5.23E-06 | 3.87E-03 | 26 | 6765 |
|  | cell part | 44464 | 3.72E-05 | 9.20E-03 | 38 | 14255 |
|  | cell | 5623 | 3.73E-05 | 9.20E-03 | 38 | 14256 |
|  | intracellular | 5622 | 6.21E-05 | 1.15E-02 | 30 | 9783 |
|  | circadian rhythm | 7623 | 9.08E-05 | 1.34E-02 | 3 | 52 |
|  | intracellular part | 44424 | 1.18E-04 | 1.37E-02 | 29 | 9547 |
|  | intracellular membrane-bounded organelle | 43231 | 1.51E-04 | 1.37E-02 | 24 | 7110 |
|  | membrane-bounded organelle | 43227 | 1.55E-04 | 1.37E-02 | 24 | 7122 |
|  | binding | 5488 | 1.77E-04 | 1.37E-02 | 30 | 10292 |
|  | transmembrane transport | 55085 | 1.86E-04 | 1.37E-02 | 7 | 723 |
|  | protein amino acid deacetylation | 6476 | 3.22E-04 | 2.14E-02 | 2 | 16 |
|  | heterocycle catabolic process | 46700 | 3.47E-04 | 2.14E-02 | 4 | 200 |
|  | cellular process | 9987 | 8.04E-04 | 4.27E-02 | 26 | 8927 |
|  | membrane | 16020 | 8.08E-04 | 4.27E-02 | 23 | 7392 |
|  | intracellular organelle | 43229 | 1.15E-03 | 4.40E-02 | 24 | 8081 |
|  | organelle | 43226 | 1.21E-03 | 4.40E-02 | 24 | 8106 |
|  | transport | 6810 | 1.32E-03 | 4.40E-02 | 11 | 2329 |
|  | establishment of localization | 51234 | 1.41E-03 | 4.40E-02 | 11 | 2348 |
|  | carboxylic acid transport | 46942 | 1.46E-03 | 4.40E-02 | 3 | 134 |
|  | rhythmic process | 48511 | 1.46E-03 | 4.40E-02 | 3 | 134 |
|  | organic acid transport | 15849 | 1.50E-03 | 4.40E-02 | 3 | 135 |
|  | anion transport | 6820 | 1.73E-03 | 4.41E-02 | 3 | 142 |
|  | mitochondrion | 5739 | 1.85E-03 | 4.55E-02 | 8 | 1376 |
|  | L-amino acid transport | 15807 | 1.94E-03 | 4.63E-02 | 2 | 39 |
| **Dataset** | **GO Term** | **GO ID** | **P value** | **Corr P value** | **# in set** | **# overall** |
| **Liver** | cation binding | 43169 | 1.58E-07 | 7.42E-05 | 19 | 3181 |
|  | ion binding | 43167 | 1.65E-07 | 7.42E-05 | 19 | 3190 |
|  | metal ion binding | 46872 | 7.32E-07 | 2.19E-04 | 18 | 3146 |
|  | catalytic activity | 3824 | 2.39E-05 | 5.36E-03 | 20 | 4858 |
|  | metabolic process | 8152 | 4.61E-05 | 8.29E-03 | 23 | 6471 |
|  | binding | 5488 | 6.06E-05 | 9.07E-03 | 30 | 10292 |
|  | S-adenosylhomocysteine metabolic process | 46498 | 1.12E-04 | 1.16E-02 | 2 | 10 |
|  | basolateral plasma membrane | 16323 | 1.20E-04 | 1.16E-02 | 4 | 158 |
|  | transition metal ion binding | 46914 | 1.32E-04 | 1.16E-02 | 10 | 1536 |
|  | intracellular part | 44424 | 1.34E-04 | 1.16E-02 | 28 | 9547 |
|  | cytoplasmic part | 44444 | 1.60E-04 | 1.16E-02 | 17 | 4194 |
|  | intracellular organelle | 43229 | 1.87E-04 | 1.16E-02 | 25 | 8081 |
|  | organelle | 43226 | 1.97E-04 | 1.16E-02 | 25 | 8106 |
|  | zinc ion binding | 8270 | 2.05E-04 | 1.16E-02 | 9 | 1313 |
|  | intracellular membrane-bounded organelle | 43231 | 2.13E-04 | 1.16E-02 | 23 | 7110 |
|  | intracellular | 5622 | 2.14E-04 | 1.16E-02 | 28 | 9783 |
|  | membrane-bounded organelle | 43227 | 2.19E-04 | 1.16E-02 | 23 | 7122 |
|  | circadian sleep/wake cycle | 42745 | 3.35E-04 | 1.68E-02 | 2 | 17 |
|  | endoplasmic reticulum | 5783 | 3.87E-04 | 1.80E-02 | 7 | 852 |
|  | leading edge membrane | 31256 | 4.21E-04 | 1.80E-02 | 2 | 19 |
|  | circadian behavior | 48512 | 4.21E-04 | 1.80E-02 | 2 | 19 |
|  | rhythmic behavior | 7622 | 5.16E-04 | 2.11E-02 | 2 | 21 |
|  | cytoplasm | 5737 | 8.83E-04 | 3.45E-02 | 21 | 6766 |
|  | hydrolase activity | 16787 | 1.47E-03 | 3.59E-02 | 10 | 2086 |
|  | focal adhesion | 5925 | 1.79E-03 | 3.92E-02 | 2 | 39 |
|  | catabolic process | 9056 | 2.00E-03 | 4.29E-02 | 6 | 830 |
|  | cell-substrate adherens junction | 5924 | 2.27E-03 | 4.75E-02 | 2 | 44 |
|  | cell part | 44464 | 2.38E-03 | 4.76E-02 | 33 | 14256 |
|  | cell | 5623 | 2.38E-03 | 4.76E-02 | 33 | 14257 |
|  | NAD or NADH binding | 51287 | 2.70E-03 | 4.79E-02 | 2 | 48 |
|  | cellular metabolic process | 44237 | 2.89E-03 | 4.79E-02 | 17 | 5371 |
|  | cell-substrate junction | 30055 | 2.92E-03 | 4.79E-02 | 2 | 50 |
|  | circadian rhythm | 7623 | 3.16E-03 | 4.79E-02 | 2 | 52 |
| **Dataset** | **GO Term** | **GO ID** | **P value** | **Corr P value** | **# in set** | **# overall** |
| **Kidney** | intracellular | 5622 | 3.64E-17 | 4.27E-14 | 45 | 9783 |
|  | cellular process | 9987 | 1.94E-16 | 8.18E-14 | 43 | 8927 |
|  | intracellular part | 44424 | 2.09E-16 | 8.18E-14 | 44 | 9547 |
|  | intracellular organelle | 43229 | 7.49E-14 | 1.34E-11 | 39 | 8081 |
|  | intracellular membrane-bounded organelle | 43231 | 8.09E-14 | 1.34E-11 | 37 | 7110 |
|  | organelle | 43226 | 8.35E-14 | 1.34E-11 | 39 | 8106 |
|  | membrane-bounded organelle | 43227 | 8.55E-14 | 1.34E-11 | 37 | 7122 |
|  | cell part | 44464 | 1.02E-13 | 1.34E-11 | 48 | 14254 |
|  | cell | 5623 | 1.02E-13 | 1.34E-11 | 48 | 14255 |
|  | cytoplasm | 5737 | 1.43E-13 | 1.68E-11 | 36 | 6766 |
|  | cytoplasmic part | 44444 | 3.11E-11 | 3.32E-09 | 27 | 4194 |
|  | cellular metabolic process | 44237 | 3.87E-11 | 3.79E-09 | 30 | 5371 |
|  | metabolic process | 8152 | 1.22E-10 | 1.10E-08 | 32 | 6470 |
|  | carboxylic acid metabolic process | 19752 | 7.42E-10 | 5.56E-08 | 11 | 518 |
|  | oxoacid metabolic process | 43436 | 7.42E-10 | 5.56E-08 | 11 | 518 |
|  | organic acid metabolic process | 6082 | 7.57E-10 | 5.56E-08 | 11 | 519 |
|  | cellular ketone metabolic process | 42180 | 9.81E-10 | 6.78E-08 | 11 | 532 |
|  | small molecule metabolic process | 44281 | 2.95E-09 | 1.92E-07 | 15 | 1350 |
|  | primary metabolic process | 44238 | 2.17E-08 | 1.34E-06 | 27 | 5586 |
|  | mitochondrion | 5739 | 3.38E-08 | 1.98E-06 | 14 | 1376 |
|  | intracellular organelle part | 44446 | 9.94E-08 | 5.56E-06 | 18 | 2625 |
|  | organelle part | 44422 | 1.53E-07 | 8.18E-06 | 18 | 2701 |
|  | fatty acid catabolic process | 9062 | 2.37E-07 | 1.21E-05 | 4 | 32 |
|  | binding | 5488 | 2.78E-07 | 1.36E-05 | 35 | 10292 |
|  | organic acid catabolic process | 16054 | 4.87E-07 | 2.20E-05 | 5 | 93 |
|  | carboxylic acid catabolic process | 46395 | 4.87E-07 | 2.20E-05 | 5 | 93 |
|  | cellular biosynthetic process | 44249 | 8.24E-07 | 3.58E-05 | 17 | 2692 |
|  | biosynthetic process | 9058 | 1.31E-06 | 5.47E-05 | 17 | 2782 |
|  | carboxylic acid binding | 31406 | 2.86E-06 | 1.13E-04 | 5 | 133 |
|  | cellular nitrogen compound metabolic process | 34641 | 2.89E-06 | 1.13E-04 | 17 | 2947 |
|  | mitochondrial membrane | 31966 | 3.09E-06 | 1.17E-04 | 7 | 380 |
|  | catalytic activity | 3824 | 3.21E-06 | 1.18E-04 | 22 | 4858 |
|  | organelle membrane | 31090 | 4.18E-06 | 1.41E-04 | 9 | 763 |
|  | nitrogen compound metabolic process | 6807 | 4.27E-06 | 1.41E-04 | 17 | 3032 |
|  | cellular lipid catabolic process | 44242 | 4.28E-06 | 1.41E-04 | 4 | 65 |
|  | mitochondrial envelope | 5740 | 4.33E-06 | 1.41E-04 | 7 | 400 |
|  | lyase activity | 16829 | 4.99E-06 | 1.58E-04 | 5 | 149 |
|  | fatty acid beta-oxidation | 6635 | 6.55E-06 | 2.02E-04 | 3 | 22 |
|  | monocarboxylic acid metabolic process | 32787 | 9.66E-06 | 2.89E-04 | 6 | 296 |
|  | cellular nitrogen compound biosynthetic process | 44271 | 9.85E-06 | 2.89E-04 | 6 | 297 |
|  | mitochondrial part | 44429 | 1.06E-05 | 3.03E-04 | 7 | 459 |
|  | cellular amino acid and derivative metabolic process | 6519 | 1.12E-05 | 3.14E-04 | 6 | 304 |
|  | lipid metabolic process | 6629 | 1.96E-05 | 5.36E-04 | 8 | 703 |
|  | organelle envelope | 31967 | 2.25E-05 | 5.85E-04 | 7 | 516 |
|  | lipid oxidation | 34440 | 2.29E-05 | 5.85E-04 | 3 | 33 |
|  | fatty acid oxidation | 19395 | 2.29E-05 | 5.85E-04 | 3 | 33 |
|  | envelope | 31975 | 2.51E-05 | 6.27E-04 | 7 | 525 |
|  | regulation of cellular metabolic process | 31323 | 2.61E-05 | 6.40E-04 | 15 | 2749 |
|  | acyl-CoA oxidase activity | 3997 | 2.71E-05 | 6.50E-04 | 2 | 5 |
|  | dicarboxylic acid metabolic process | 43648 | 3.52E-05 | 8.27E-04 | 3 | 38 |
|  | macromolecular complex | 32991 | 3.84E-05 | 8.83E-04 | 14 | 2493 |
|  | glutamine family amino acid metabolic process | 9064 | 4.44E-05 | 1.00E-03 | 3 | 41 |
|  | monocarboxylic acid binding | 33293 | 5.12E-05 | 1.14E-03 | 3 | 43 |
|  | regulation of biological quality | 65008 | 5.22E-05 | 1.14E-03 | 10 | 1315 |
|  | cellular amino acid biosynthetic process | 8652 | 5.49E-05 | 1.17E-03 | 3 | 44 |
|  | regulation of metabolic process | 19222 | 5.60E-05 | 1.17E-03 | 15 | 2934 |
|  | cellular amine metabolic process | 44106 | 6.36E-05 | 1.30E-03 | 5 | 253 |
|  | small molecule biosynthetic process | 44283 | 6.41E-05 | 1.30E-03 | 6 | 415 |
|  | regulation of primary metabolic process | 80090 | 6.76E-05 | 1.34E-03 | 14 | 2625 |
|  | lipid catabolic process | 16042 | 8.85E-05 | 1.73E-03 | 4 | 140 |
|  | urea cycle | 50 | 9.72E-05 | 1.84E-03 | 2 | 9 |
|  | urea metabolic process | 19627 | 9.72E-05 | 1.84E-03 | 2 | 9 |
|  | amide biosynthetic process | 43604 | 1.21E-04 | 2.23E-03 | 2 | 10 |
|  | oxidoreductase activity, acting on the CH-CH group of donors, oxygen as acceptor | 16634 | 1.21E-04 | 2.23E-03 | 2 | 10 |
|  | mitochondrial inner membrane | 5743 | 1.46E-04 | 2.56E-03 | 5 | 302 |
|  | glutamate metabolic process | 6536 | 1.48E-04 | 2.56E-03 | 2 | 11 |
|  | arginine metabolic process | 6525 | 1.48E-04 | 2.56E-03 | 2 | 11 |
|  | oxaloacetate metabolic process | 6107 | 1.48E-04 | 2.56E-03 | 2 | 11 |
|  | organelle inner membrane | 19866 | 1.75E-04 | 2.98E-03 | 5 | 314 |
|  | lipid modification | 30258 | 2.11E-04 | 3.54E-03 | 3 | 69 |
|  | amine metabolic process | 9308 | 2.33E-04 | 3.85E-03 | 5 | 334 |
|  | amine biosynthetic process | 9309 | 2.39E-04 | 3.88E-03 | 3 | 72 |
|  | glutamine family amino acid biosynthetic process | 9084 | 2.44E-04 | 3.88E-03 | 2 | 14 |
|  | acyl-CoA dehydrogenase activity | 3995 | 2.44E-04 | 3.88E-03 | 2 | 14 |
|  | cellular amino acid metabolic process | 6520 | 2.63E-04 | 4.12E-03 | 4 | 186 |
|  | cellular amide metabolic process | 43603 | 2.82E-04 | 4.34E-03 | 2 | 15 |
|  | regulation of cellular biosynthetic process | 31326 | 2.85E-04 | 4.34E-03 | 12 | 2278 |
|  | microtubule cytoskeleton | 15630 | 3.04E-04 | 4.58E-03 | 5 | 354 |
|  | regulation of biosynthetic process | 9889 | 3.11E-04 | 4.62E-03 | 12 | 2300 |
|  | small molecule catabolic process | 44282 | 3.25E-04 | 4.76E-03 | 5 | 359 |
|  | fatty acid metabolic process | 6631 | 3.47E-04 | 5.03E-03 | 4 | 200 |
|  | cellular macromolecule metabolic process | 44260 | 4.02E-04 | 5.76E-03 | 16 | 3903 |
|  | biological regulation | 65007 | 4.62E-04 | 6.54E-03 | 23 | 7123 |
|  | gluconeogenesis | 6094 | 5.60E-04 | 7.83E-03 | 2 | 21 |
|  | glycerol metabolic process | 6071 | 6.15E-04 | 8.48E-03 | 2 | 22 |
|  | developmental process | 32502 | 6.21E-04 | 8.48E-03 | 13 | 2855 |
|  | cellular catabolic process | 44248 | 6.32E-04 | 8.52E-03 | 6 | 635 |
|  | oxidation reduction | 55114 | 6.58E-04 | 8.78E-03 | 6 | 640 |
|  | microtubule | 5874 | 6.78E-04 | 8.94E-03 | 4 | 239 |
|  | regulation of macromolecule metabolic process | 60255 | 7.29E-04 | 9.50E-03 | 12 | 2527 |
|  | hexose biosynthetic process | 19319 | 7.96E-04 | 9.97E-03 | 2 | 25 |
|  | alditol metabolic process | 19400 | 7.96E-04 | 9.97E-03 | 2 | 25 |
|  | nucleus | 5634 | 7.99E-04 | 9.97E-03 | 16 | 4149 |
|  | microbody | 42579 | 8.07E-04 | 9.97E-03 | 3 | 109 |
|  | peroxisome | 5777 | 8.07E-04 | 9.97E-03 | 3 | 109 |
|  | oxidoreductase activity | 16491 | 8.81E-04 | 1.08E-02 | 6 | 677 |
|  | homeostatic process | 42592 | 9.51E-04 | 1.15E-02 | 6 | 687 |
|  | gene expression | 10467 | 1.12E-03 | 1.34E-02 | 11 | 2281 |
|  | multicellular organismal development | 7275 | 1.15E-03 | 1.36E-02 | 12 | 2660 |
|  | pyruvate metabolic process | 6090 | 1.31E-03 | 1.53E-02 | 2 | 32 |
|  | cellular lipid metabolic process | 44255 | 1.35E-03 | 1.54E-02 | 5 | 493 |
|  | protein complex | 43234 | 1.46E-03 | 1.54E-02 | 10 | 1995 |
|  | fatty acid binding | 5504 | 1.48E-03 | 1.54E-02 | 2 | 34 |
|  | monosaccharide biosynthetic process | 46364 | 1.48E-03 | 1.54E-02 | 2 | 34 |
|  | anatomical structure development | 48856 | 1.49E-03 | 1.54E-02 | 11 | 2363 |
|  | macromolecule metabolic process | 43170 | 1.52E-03 | 1.54E-02 | 16 | 4401 |
|  | negative regulation of apoptosis | 43066 | 1.59E-03 | 1.54E-02 | 4 | 301 |
|  | negative regulation of programmed cell death | 43069 | 1.69E-03 | 1.55E-02 | 4 | 306 |
|  | soluble fraction | 5625 | 1.81E-03 | 1.65E-02 | 4 | 312 |
|  | protein binding | 5515 | 1.83E-03 | 1.66E-02 | 18 | 5384 |
|  | negative regulation of cell death | 60548 | 1.90E-03 | 1.70E-02 | 4 | 316 |
|  | organic acid biosynthetic process | 16053 | 2.02E-03 | 1.78E-02 | 3 | 150 |
|  | carboxylic acid biosynthetic process | 46394 | 2.02E-03 | 1.78E-02 | 3 | 150 |
|  | hydro-lyase activity | 16836 | 2.04E-03 | 1.79E-02 | 2 | 40 |
|  | cellular macromolecule biosynthetic process | 34645 | 2.13E-03 | 1.85E-02 | 10 | 2099 |
|  | triglyceride metabolic process | 6641 | 2.14E-03 | 1.85E-02 | 2 | 41 |
|  | multicellular organismal process | 32501 | 2.18E-03 | 1.87E-02 | 16 | 4553 |
|  | macromolecule biosynthetic process | 9059 | 2.21E-03 | 1.88E-02 | 10 | 2109 |
|  | polyol metabolic process | 19751 | 2.46E-03 | 2.08E-02 | 2 | 44 |
|  | catabolic process | 9056 | 2.48E-03 | 2.08E-02 | 6 | 830 |
|  | system development | 48731 | 2.55E-03 | 2.11E-02 | 10 | 2151 |
|  | oxidoreductase activity, acting on the CH-CH group of donors | 16627 | 2.57E-03 | 2.11E-02 | 2 | 45 |
|  | alcohol biosynthetic process | 46165 | 2.57E-03 | 2.11E-02 | 2 | 45 |
|  | non-membrane-bounded organelle | 43228 | 2.62E-03 | 2.12E-02 | 9 | 1798 |
|  | intracellular non-membrane-bounded organelle | 43232 | 2.62E-03 | 2.12E-02 | 9 | 1798 |
|  | regulation of macromolecule biosynthetic process | 10556 | 2.68E-03 | 2.15E-02 | 10 | 2166 |
|  | coenzyme binding | 50662 | 2.69E-03 | 2.15E-02 | 3 | 166 |
|  | structural molecule activity | 5198 | 2.77E-03 | 2.20E-02 | 4 | 351 |
|  | negative regulation of cellular metabolic process | 31324 | 2.89E-03 | 2.27E-02 | 5 | 587 |
|  | acylglycerol metabolic process | 6639 | 2.92E-03 | 2.27E-02 | 2 | 48 |
|  | cellular response to insulin stimulus | 32869 | 3.04E-03 | 2.27E-02 | 2 | 49 |
|  | regulation of protein amino acid phosphorylation | 1932 | 3.07E-03 | 2.27E-02 | 3 | 174 |
|  | neutral lipid metabolic process | 6638 | 3.17E-03 | 2.27E-02 | 2 | 50 |
|  | glycerol ether metabolic process | 6662 | 3.17E-03 | 2.27E-02 | 2 | 50 |
|  | regulation of nucleobase, nucleoside, nucleotide and nucleic acid metabolic process | 19219 | 3.26E-03 | 2.27E-02 | 10 | 2225 |
|  | alcohol metabolic process | 6066 | 3.38E-03 | 2.29E-02 | 4 | 371 |
|  | carbon-oxygen lyase activity | 16835 | 3.42E-03 | 2.29E-02 | 2 | 52 |
|  | organic ether metabolic process | 18904 | 3.42E-03 | 2.29E-02 | 2 | 52 |
|  | regulation of transcription, DNA-dependent | 6355 | 3.44E-03 | 2.29E-02 | 7 | 1192 |
|  | regulation of phosphorylation | 42325 | 3.45E-03 | 2.29E-02 | 4 | 373 |
|  | regulation of nitrogen compound metabolic process | 51171 | 3.56E-03 | 2.35E-02 | 10 | 2252 |
|  | cellular response to peptide hormone stimulus | 71375 | 3.68E-03 | 2.42E-02 | 2 | 54 |
|  | regulation of RNA metabolic process | 51252 | 3.77E-03 | 2.46E-02 | 7 | 1212 |
|  | positive regulation of cell proliferation | 8284 | 3.89E-03 | 2.53E-02 | 4 | 386 |
|  | mRNA binding | 3729 | 3.96E-03 | 2.53E-02 | 2 | 56 |
|  | regulation of phosphorus metabolic process | 51174 | 3.97E-03 | 2.53E-02 | 4 | 388 |
|  | regulation of phosphate metabolic process | 19220 | 3.97E-03 | 2.53E-02 | 4 | 388 |
|  | regulation of apoptosis | 42981 | 4.17E-03 | 2.65E-02 | 5 | 640 |
|  | nucleic acid metabolic process | 90304 | 4.38E-03 | 2.77E-02 | 10 | 2319 |
|  | regulation of programmed cell death | 43067 | 4.42E-03 | 2.78E-02 | 5 | 649 |
|  | nucleobase, nucleoside, nucleotide and nucleic acid metabolic process | 6139 | 4.46E-03 | 2.78E-02 | 11 | 2718 |
|  | transmembrane receptor protein tyrosine kinase signaling pathway | 7169 | 4.54E-03 | 2.78E-02 | 3 | 200 |
|  | regulation of peptidyl-tyrosine phosphorylation | 50730 | 4.68E-03 | 2.78E-02 | 2 | 61 |
|  | cellular carbohydrate biosynthetic process | 34637 | 4.68E-03 | 2.78E-02 | 2 | 61 |
|  | negative regulation of metabolic process | 9892 | 4.69E-03 | 2.78E-02 | 5 | 658 |
|  | regulation of cell death | 10941 | 4.81E-03 | 2.78E-02 | 5 | 662 |
|  | regulation of transcription | 45449 | 4.97E-03 | 2.78E-02 | 9 | 1980 |
|  | cofactor metabolic process | 51186 | 5.00E-03 | 2.78E-02 | 3 | 207 |
|  | negative regulation of biological process | 48519 | 5.08E-03 | 2.81E-02 | 8 | 1623 |
|  | regulation of transcription from RNA polymerase II promoter | 6357 | 5.15E-03 | 2.84E-02 | 5 | 673 |
|  | positive regulation of cellular process | 48522 | 5.38E-03 | 2.95E-02 | 8 | 1639 |
|  | regulation of protein modification process | 31399 | 5.69E-03 | 3.10E-02 | 3 | 217 |
|  | FAD binding | 50660 | 5.78E-03 | 3.10E-02 | 2 | 68 |
|  | site of polarized growth | 30427 | 5.78E-03 | 3.10E-02 | 2 | 68 |
|  | growth cone | 30426 | 5.78E-03 | 3.10E-02 | 2 | 68 |
|  | ribonucleoprotein complex | 30529 | 5.79E-03 | 3.10E-02 | 4 | 432 |
|  | amine catabolic process | 9310 | 5.94E-03 | 3.17E-02 | 2 | 69 |
|  | nucleotide binding | 166 | 6.09E-03 | 3.23E-02 | 9 | 2043 |
|  | regulation of kinase activity | 43549 | 6.44E-03 | 3.28E-02 | 3 | 227 |
|  | response to insulin stimulus | 32868 | 6.46E-03 | 3.28E-02 | 2 | 72 |
|  | transcription coactivator activity | 3713 | 6.81E-03 | 3.34E-02 | 2 | 74 |
|  | cofactor binding | 48037 | 6.84E-03 | 3.35E-02 | 3 | 232 |
|  | regulation of transferase activity | 51338 | 7.09E-03 | 3.45E-02 | 3 | 235 |
|  | receptor binding | 5102 | 7.46E-03 | 3.62E-02 | 5 | 736 |
|  | regulation of response to stress | 80134 | 7.85E-03 | 3.78E-02 | 3 | 244 |
|  | regulation of secretion | 51046 | 7.85E-03 | 3.78E-02 | 3 | 244 |
|  | regulation of biological process | 50789 | 8.76E-03 | 3.97E-02 | 19 | 6696 |
|  | generation of precursor metabolites and energy | 6091 | 8.76E-03 | 3.97E-02 | 3 | 254 |
|  | regulation of cellular response to stress | 80135 | 9.10E-03 | 4.08E-02 | 2 | 86 |
|  | mammary gland development | 30879 | 9.10E-03 | 4.08E-02 | 2 | 86 |
|  | negative regulation of cellular biosynthetic process | 31327 | 9.13E-03 | 4.08E-02 | 4 | 493 |
|  | mRNA processing | 6397 | 9.14E-03 | 4.08E-02 | 3 | 258 |
|  | negative regulation of cellular process | 48523 | 9.44E-03 | 4.20E-02 | 7 | 1439 |
|  | carbohydrate biosynthetic process | 16051 | 9.51E-03 | 4.21E-02 | 2 | 88 |
|  | translation | 6412 | 9.53E-03 | 4.21E-02 | 3 | 262 |
|  | negative regulation of biosynthetic process | 9890 | 9.58E-03 | 4.21E-02 | 4 | 500 |
|  | multicellular organismal homeostasis | 48871 | 9.92E-03 | 4.22E-02 | 2 | 90 |
|  | cellular response to hormone stimulus | 32870 | 1.04E-02 | 4.37E-02 | 2 | 92 |
|  | regulation of gene expression | 10468 | 1.06E-02 | 4.44E-02 | 9 | 2229 |
|  | cellular response to endogenous stimulus | 71495 | 1.10E-02 | 4.60E-02 | 2 | 95 |
|  | positive regulation of biological process | 48518 | 1.10E-02 | 4.60E-02 | 8 | 1854 |
|  | cytoskeleton | 5856 | 1.12E-02 | 4.66E-02 | 6 | 1137 |
|  | regulation of cellular process | 50794 | 1.17E-02 | 4.74E-02 | 18 | 6371 |
|  | membrane | 16020 | 1.18E-02 | 4.76E-02 | 20 | 7392 |
|  | enzyme linked receptor protein signaling pathway | 7167 | 1.20E-02 | 4.82E-02 | 3 | 285 |
|  | regulation of blood pressure | 8217 | 1.24E-02 | 4.88E-02 | 2 | 101 |
|  | sulfur metabolic process | 6790 | 1.24E-02 | 4.88E-02 | 2 | 101 |
|  | energy derivation by oxidation of organic compounds | 15980 | 1.24E-02 | 4.88E-02 | 2 | 101 |
|  | nucleotidyltransferase activity | 16779 | 1.26E-02 | 4.88E-02 | 2 | 102 |
|  | cytoskeleton organization | 7010 | 1.30E-02 | 4.88E-02 | 3 | 294 |
|  | metal ion binding | 46872 | 1.30E-02 | 4.88E-02 | 11 | 3146 |
|  | transition metal ion binding | 46914 | 1.32E-02 | 4.88E-02 | 7 | 1536 |
|  | mRNA metabolic process | 16071 | 1.32E-02 | 4.88E-02 | 3 | 296 |
|  | structural constituent of ribosome | 3735 | 1.33E-02 | 4.89E-02 | 2 | 105 |

**Table S3.** Sequences of primers used in this study. All sequences are for human genes. The control mTERF, POLRMT, TFB2M, mTERF3, and β-actin ChIP sequences are from Bruni et al. (2010).

| **Gene** | **Purpose** | **Direction** | **Sequence** |
| --- | --- | --- | --- |
| SIRT3 | qPCR | Forward | 5’-AGCCCTCTTCATGTTCCGAAGTGT-3’ |
|  |  | Reverse | 5’-TCATGTCAACACCTGCAGTCCCTT-3’ |
| NRF-2α | qPCR | Forward | 5’-GGCGCGTAGGTTTGTTCTAC-3’ |
|  |  | Reverse | 5’-ACTCCAGCCATGACTAAAAGAGA-3’ |
| NRF-2β1 | qPCR | Forward | 5’-ACCAACCAGTGGGATGGGTCAG-3’ |
|  |  | Reverse | 5’-GCACATTCCACCCGGCTCTCAAT-3’ |
| POLG2 | qPCR | Forward | 5’-AAGGTTGCTTTGGATGTAGGAAGA-3’ |
|  |  | Reverse | 5’-GGCCACACAGAAATCCCATT-3’ |
| B2M | qPCR | Forward | 5’-AGATGAGTATGCCTGCCGTGTGAA-3’ |
|  |  | Reverse | 5’-TGCTGCTTACATGTCTCGATCCCA-3’ |
| SIRT3 | ChIP | Forward | 5’-CATGACAGCAGGAAGACCCC-3’ |
|  |  | Reverse | 5’-CAAACGCCGGAGAGTTTTGT-3’ |
| mTERF | ChIP | Forward | 5’-GACCAACGACATCACCTCTGC-3’ |
|  |  | Reverse | 5’-CACCCATCCACTGTAGTTCGC-3’ |
| POLRMT | ChIP | Forward | 5’-AAAACAGCAGGAGGAACCAATC-3’ |
|  |  | Reverse | 5’-CCGGGAGTTGTGGTTTCATG-3’ |
| TFB2M | ChIP | Forward | 5’-GGTCGGTCGCTCTCCTCAA-3’ |
|  |  | Reverse | 5’-AAACACTAGAGCCTGCGCATG-3’ |
| mTERF3 | ChIP | Forward | 5’-CTGTCTCCCCGCGTAACC-3’ |
|  |  | Reverse | 5’-CTCCTCAGCCCGCCCTAC-3’ |
| β-actin | ChIP | Forward | 5’-CCCAGCCATGTACGTTGCTA-3’ |
|  |  | Reverse | 5’-CGTCACCGGAGTCCATCAC-3’ |
| NRF-2 (DM 1) | Mutagenesis | Forward | 5’-CGGAAATGCTCACTCACTTAAGGCGCCGAGCG-3’ |
|  |  | Reverse | 5’-CGCTCGGCGCCTTAAGTGAGTGAGCATTTCCG-3’ |
| NRF-2 (DM 2) | Mutagenesis | Forward | 5’- CGCGGGGATGGCTGCCTTAAATGCTCACTCACTT-3’ |
|  |  | Reverse | 5’- AAGTGAGTGAGCATTTAAGGCAGCCATCCCCGCG-3’ |
| NRF-2 (DEL) | Mutagenesis | Forward | 5’-GCCGAGCGGCGCGGGGCA-3’ |
|  |  | Reverse | 5’-AGCCATCCCCGCGGTGCTGACATC-3’ |
