# Supplementary material for: Nuclear respiratory factor 2 induces SIRT3 expression
Source: Aging Cell. 2015 Jun 24;14(5):818–25. doi: 10.1111/acel.12360 (PMC4568969; doi:10.1111/acel.12360)
Supplement: Supplementary file 1 [file acel0014-0818-sd1.pdf]

Figure S1

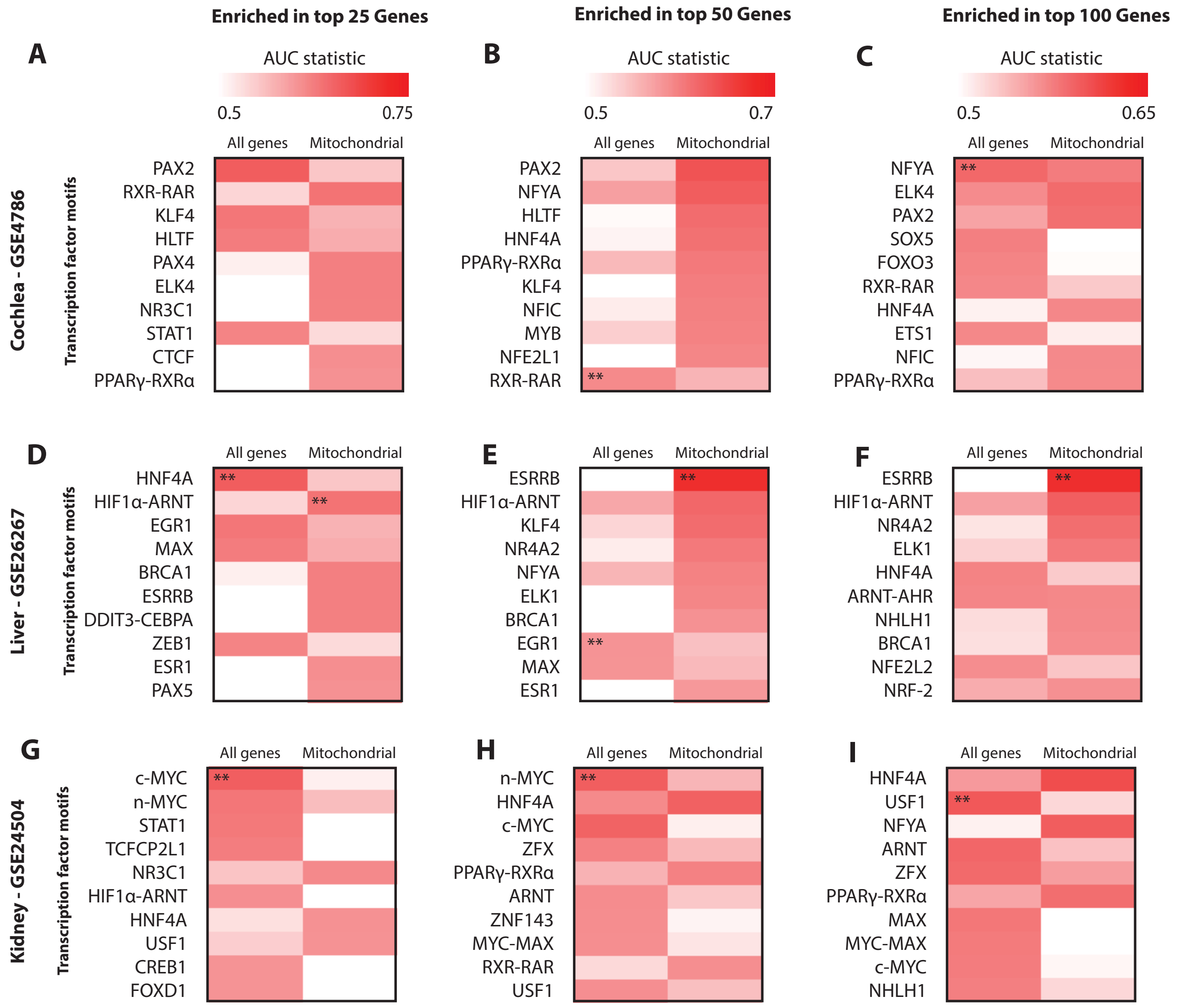

Enriched in top 25 Genes
Enriched in top 50 Genes
Enriched in top 100 Genes
A
B
C
AUC statistic
0.5
0.75
0.7
0.65
All genes
Mitochondrial
Cochlea - GSE4786
Transcription factor motifs
PAX2
RXR-RAR
KLF4
HLTF
PAX4
ELK4
NR3C1
STAT1
CTCF
PPARγ-RXRα
NFYA
HNF4A
NFIC
MYB
NFE2L1
ELK4
SOX5
FOXO3
ETS1
**
D
E
F
Liver - GSE26267
HNF4A
HIF1α-ARNT
EGR1
MAX
BRCA1
ESRRB
DDIT3-CEBPA
ZEB1
ESR1
PAX5
NR4A2
NFYA
ELK1
ARNT-AHR
NHLH1
NFE2L2
NRF-2
G
H
I
Kidney - GSE24504
c-MYC
n-MYC
STAT1
TCFCP2L1
NR3C1
USF1
CREB1
FOXD1
ZFX
ARNT
ZNF143
MYC-MAX
RXR-RAR
